# Diagnostic utility of corneal confocal microscopy and intra-epidermal nerve fibre density in diabetic neuropathy

**DOI:** 10.1371/journal.pone.0180175

**Published:** 2017-07-18

**Authors:** Uazman Alam, Maria Jeziorska, Ioannis N. Petropoulos, Omar Asghar, Hassan Fadavi, Georgios Ponirakis, Andrew Marshall, Mitra Tavakoli, Andrew J. M. Boulton, Nathan Efron, Rayaz A. Malik

**Affiliations:** 1 Diabetes & Endocrinology Research, Department of Eye & Vision Sciences, Institute of Ageing and Chronic Disease, University of Liverpool and Aintree University Hospital NHS Foundation Trust, Liverpool, United Kingdom; 2 Division of Diabetes, Endocrinology and Gastroenterology, Institute of Human Development, University of Manchester and the Manchester Royal Infirmary, Central Manchester Hospital Foundation Trust, Manchester, United Kingdom; 3 Weill Cornell Medicine-Qatar, Doha, Qatar; 4 University of Exeter, Exeter, United Kingdom; 5 Institute of Health and Biomedical Innovation, Queensland University of Technology, Brisbane, Queensland, Australia; Hirosaki Daigaku, JAPAN

## Abstract

**Objectives:**

Corneal confocal microscopy (CCM) is a rapid, non-invasive, reproducible technique that quantifies small nerve fibres. We have compared the diagnostic capability of CCM against a range of established measures of nerve damage in patients with diabetic neuropathy.

**Methods:**

In this cross sectional study, thirty subjects with Type 1 diabetes without neuropathy (T1DM), thirty one T1DM subjects with neuropathy (DSPN) and twenty seven non-diabetic healthy control subjects underwent detailed assessment of neuropathic symptoms and neurologic deficits, quantitative sensory testing (QST), electrophysiology, skin biopsy and corneal confocal microscopy (CCM).

**Results:**

Subjects with DSPN were older (C vs T1DM vs DSPN: 41.0±14.9 vs 38.8±12.5 vs 53.3±11.9, P = 0.0002), had a longer duration of diabetes (P<0.0001), lower eGFR (P = 0.006) and higher albumin-creatinine ratio (P = 0.03) with no significant difference for HbA1c, BMI, lipids and blood pressure. Patients with DSPN were representative of subjects with diabetic neuropathy with clinical signs and symptoms of neuropathy and greater neuropathy deficits quantified by QST, electrophysiology, intra-epidermal nerve fibre density and CCM. Corneal nerve fibre density (CNFD) (Spearman’s Rho = 0.60 P<0.0001) and IENFD (Spearman’s Rho = 0.56 P<0.0001) were comparable when correlated with peroneal nerve conduction velocity. For the diagnosis of diabetic neuropathy the sensitivity for CNFD was 0.77 and specificity was 0.79 with an area under the ROC curve of 0.81. IENFD had a diagnostic sensitivity of 0.61, specificity of 0.80 and area under the ROC curve of 0.73.

**Conclusions:**

CCM is a valid accurate non-invasive method to identify small nerve fibre pathology and is able to diagnose DPN.

## Introduction

Diabetic peripheral neuropathy is a debilitating condition which may lead to pain, foot ulceration and eventual amputation. Therefore it is important to accurately diagnose both early diabetic neuropathy and those at high risk of foot ulceration [1]. A series of recent detailed studies have shown that the clinical neurological assessment has poor reproducibility [2], quantitative sensory testing (QST) showed good reproducibility, but remains subjective [3]. Nerve conduction studies are advocated as an essential component for the diagnosis of diabetic neuropathy [4], however, small nerve fibres are the earliest to degenerate [5] and regenerate [6] and indeed are central to the genesis of pain and development of foot ulceration [7].

In 2005 the European Federation of Neurological Societies published guidelines on the use of skin biopsy in the diagnosis of peripheral neuropathies [8] and more recently the value of the technique has been further emphasized [9]. Currently, skin biopsy with an assessment of intra-epidermal nerve fibres (IENF) is considered the gold standard for the evaluation of small fibre neuropathy and has been advocated for use as a measure of treatment response in clinical trials [10]. Previously skin biopsy has demonstrated a good diagnostic ability of for small fibre neuropathy [11–13]. Hence reproducible and reliable processing and accurate quantification methods have been established for assessing IENF pathology against normative ranges [14]. However, despite being advocated as an endpoint in clinical trials of diabetic neuropathy there is surprisingly scarce data which have established the diagnostic ability of skin biopsy for diabetic neuropathy [7]. Furthermore skin biopsy is invasive with a small but significant risk for bleeding and infection and requires expertise in laboratory assessment.

CCM is a non-invasive ophthalmic application, which is rapid, non-invasive and readily reproducible for quantifying small nerve fibres and has been shown to diagnose and track the progression of diabetic neuropathy [15]. CCM has been shown to have reasonable diagnostic utility in detecting DPN diagnosed using NDS [16] and has good reproducibility [17]. More recently CCM has been shown to correlate with functional measures of small nerve fibre injury [18] and indeed has been shown to precede an abnormality in neurophysiology in patients with T1DM [19]. However, very few studies have directly assessed CCM against currently accepted gold standard FDA approved methods such as skin biopsy, QST and nerve conduction studies in the diagnosis of diabetic neuropathy. We have therefore compared the ability of CCM, skin biopsy and QST in the diagnosis of diabetic neuropathy using the Toronto criteria for definite diabetic neuropathy (presence of an abnormality of nerve conduction and a symptom or symptoms or a sign or signs of neuropathy) [4].

## Research design and methods

### Selection of patients

In this cross sectional study, thirty subjects with Type 1 diabetes without neuropathy (T1DM) (n = 30), thirty one T1DM subjects with neuropathy (DSPN) (n = 31) and twenty seven non-diabetic healthy control subjects (Controls) (n = 27) were evaluated. Subjects were consecutively assessed and were unselected. Subjects with a history of neurologic conditions, ocular trauma or previous ocular surgery were excluded. Subjects were assessed at the Wellcome Trust Clinical Research Facility (Manchester) from March 2010 to May 2013 and were not recruited base on symptomatology of painful neuropathy. The study population was recruited from a consecutive series of participants defined by the selection criteria. Data collection was planned prospectively. The study was approved by the North Manchester Research Ethics committee, and written informed consent was obtained according to the Declaration of Helsinki. Funding was provided by JDRF, this study was investigator led. All diagnostic tests were carried out in the same set of visit(s) with neurophysiology occasionally carried out on set days. Intervals between tests were minimal with no active intervention administered in this study.

### Definition of neuropathy

Diabetic neuropathy was defined according to the Toronto criteria by the presence of an abnormality of nerve conduction and a symptom or symptoms or a sign or signs of neuropathy [4]. Nerve conduction studies, were carried out by a consultant neurophysiologist.

### Assessment of neuropathy

All patients and control subjects underwent a detailed evaluation of neurologic symptoms by a qualified physician according to the neuropathy symptom profile (NSP), and the McGill VAS was used to assess the severity of painful neuropathy. Clinical neurologic deficits were assessed using the modified neuropathy disability score, which includes an evaluation of vibration, pin prick, and temperature perception as well as the presence or absence of ankle reflexes. Quantitative sensory testing included an assessment of the vibration perception threshold (VPT), measured using a neurothesiometer (Horwell, Scientific Laboratory Supplies, Wilford, Nottingham, U.K.), cold sensation (CST) (Aδ fibres) and warm sensation (WST) (c fibres) thresholds using the method of limits with the MEDOC TSA II (Medoc, Ramat Vishay, Israel) on the dorsum of the left foot. CASE IV was used to measure the heart rate response to deep breathing (HR-DVB) over two 8-cycle breathing series separated by a 5-min period of normal breathing. Electro-diagnostic studies were undertaken using a Dante “Key point” system (Dante Dynamics, Bristol, U.K.) equipped with a DISA temperature regulator to keep limb temperature constantly between 32°C and 35°C. Peroneal motor and sural sensory nerves were assessed in the right lower limb by a consultant neurophysiologist. The motor study was performed using silver- silver chloride surface electrodes at standardized sites defined by anatomical landmarks, and recordings for the sural nerve were taken using antidromic stimulation over a distance of 100 mm. Neuropathy status of the index subject were unknown at the time of the above assessments.

### Corneal confocal microscopy

Patients underwent examination with the Heidelberg retina tomography III in vivo corneal confocal microscope employing our established methodology for image acquisition [20] by two qualified optometrists (INP and MT) and only one optometrist (INP) undertook analysis of corneal nerve morphology. We have previously shown good intra- and inter-observer repeatability for quantification of corneal nerve morphology using CCM [17]. Several scans of the entire depth of the cornea were recorded by turning the fine focus of the objective lens backward and forward for ~2 min using the section mode, which enables manual acquisition and storage of single images of all corneal layers. This provides en face two-dimensional images with a lateral resolution of ~2 mm/pixel and final image size of 400 x 400 pixels of the sub-basal nerve plexus of the cornea from each patient and control subject. Each sub-basal nerve fibre bundle contains unmyelinated fibres, which run parallel to Bowman’s layer before dividing and terminating as individual axons underneath the surface epithelium. Five images per patient from the centre of the cornea were selected and examined in a masked and randomized fashion [21]. Three corneal nerve parameters were quantified: 1) CNFD, the total number of major nerves per square millimetre of corneal tissue (no.mm^2^); 2) corneal nerve branch density (CNBD), the number of branches emanating from all major nerve trunks per square millimetre of corneal tissue (no.mm^2^); and 3) corneal nerve fibre length (CNFL), the total length of all nerve fibres and branches (mm/mm^2^) within the area of corneal tissue. Only one healthy control and one subject with diabetes did not complete CCM assessment in this study. Quantification of corneal nerve parameters was undertaken in a blinded fashion.

### Skin biopsy and immunohistochemistry

A sub-cohort of participants underwent a 3-mm punch skin biopsy from the dorsum of the foot; 2 cm above the second metatarsal head after local anaesthesia (1% lidocaine). The biopsy site was closed using Steristrips, and the specimen was immediately fixed in PBS-buffered 4% paraformaldehyde for 18–24 h, washed—in Tris-buffered saline, cryoprotected in sucrose, frozen in liquid nitrogen and stored at -80°C- and subsequently cut into 50-μm sections on a cryostat microtome. Five floating sections per subject were immunostained for PGP9.5 neuronal marker. Non-specific protein binding and endogenous peroxidase activity were blocked by incubation in 5% goat serum and 0.3% hydrogen peroxide, respectively. The anti-PGP9.5 antibody (EMD Milipore, Billerica, MA, USA) was followed first by goat anti-rabbit IgG and then by HRP-Streptavidin (both diluted 1:1000, both from Vector Laboratories, Peterborough, UK). Nerve fibres were visualised by SG chromogen (Vector Laboratories). Intraepidermal nerve fibre density (IENFD) was calculated as the number of nerve fibres crossing the basement membrane of the epidermis and expressed per millimetre length of epidermis. Analysis of the IENF was conducted in a blind fashion by two assessors and all subjects completed skin biopsy assessment.

### Statistical analysis

Statistical analyses were undertaken on Statsdirect (Statsdirect, Cheshire, UK). The data are expressed as Mean ± standard deviation (SD). ANOVA method or a non-parametric counterpart, Kruskal-Wallis were used to assess differences between groups depending on normality of the data. The Mann-Whitney U test was used to compare T1DM with DSPN for the duration of diabetes. Chi squared analyses were used to assess frequencies of gender, and ethnicity. Overall the *p* value was maintained at 0.05 for multiple comparison tests (Bonferroni adjustment or Conover-Inmann pairwise comparison). Spearman’s rank correlation was undertaken for CNFD, CNBD, CNFL and IENFD versus NDS, McGill VAS, NSP, IENFD, thermal thresholds, VPT, and nerve conduction studies. ROC curve analyses were used to define the Wilcoxon estimate of area under ROC curve, optimal cut offs with associated sensitivity and specificity for CCM parameters, IENFD, VPT and thermal thresholds. Positive predictive value (PPV) and negative predictive value (NPV) were calculated for the three diagnostic measures, which had the greatest Wilcoxon estimate of area under the ROC curve.

## Results

### Demographics, metabolic and anthropometric assessment (Table 1)

We report no adverse events from this study. The participant demographics and metabolic and anthropometric measurements in diabetic patients and control subjects are summarized in Table 1. Patients with DSPN (53.3±11.9) were significantly older than controls (41.0±14.9 years, P = 0.0008) and T1DM without DSPN (38.8±12.5, P<0.0001) and the duration of diabetes was greater in those with DSPN compared to those without DSPN (P<0.0001). HbA1c (P<0.0001) was significantly higher in diabetic patients compared with control subjects with no difference between T1DM patients with and without DSPN. Total cholesterol was significantly lower in diabetic patients without (P = 0.006) and with (P = 0.002) DSPN compared to control subjects. BMI, HDL, triglycerides, systolic and diastolic blood pressure were comparable between diabetic patients and control subjects. The estimated glomerular filtration rate was lower in DSPN compared to T1DM without DSPN (P = 0.006), the Albumin-Creatinine Ratio (ACR) was higher in DSPN compared to T1DM without DSPN (P = 0.03) and controls (P = 0.004), although median values for controls, T1DM without DSPN and with DSPN were all within the normal range.

**Table 1:** Participant demographics and metabolic parameters in control subjects and diabetic patients without (T1DM) and with (DSPN) neuropathy, with statistically significant differences between groups.

	C (n = 27)	T1DM (n = 30)	DSPN (n = 31)	*T1DM v DSPN*
Age (years)	41.0±14.9	38.8±12.5	53.3±11.9	0.0002
**Gender (Male) (%)**	**59**	**43**	**61**	**-**
**Ethnicity (White European) (%)**	**74**	**90**	**97**	**-**
**Aetiology of Diabetes (Type 1 DM) (%)**	-	100	100	NS
**Duration of Diabetes (years)**	**-**	**17.2±12.0**	**37.2±13.1**	**<0.0001**
**HbA1c (%)**	5.5±0.3	8.0±1.3	8.5±1.5	NS
**HbA1c (mmol/mol)**	36.9±3.4	61.0±21.0	70.0±17.0	NS
**BMI (kg/m2)**	26.9±4.0	26.3±4.4	27.2±4.2	NS
**T-CHL (mmol/l)**	5.0±0.8	4.4±0.9	4.3±0.9	NS
**HDL-C (mmol/l)**	1.6±0.4	1.6±0.4	1.6±0.5	NS
**Triglycerides**	1.3±0.6	1.2±0.8	1.3±0.7	NS
**Systolic BP (mmHg)**	128±18	126±17	132±22	NS
**Diastolic BP (mmHg)**	70±10	71±10	72±9	NS
**ACR (mg/mmol)**	**0.4±0.4**	**0.7±0.9**	**2.8±4.8**	**0.03**
**Median (IQR)**	**0.3 (0.2–0.4)**	**0.3 (0.2–0.5)**	**0.7 (0.2–2.7)**	
**eGFR (ml/min/1.73)**	**85±7**	**90±3**	**80±18ƒ**	**0.006**

Post Hoc Analyses

Age C vs T1DM (NS), C vs DSPN (P = 0.0008), T1DM vs DSPN (P<0.0001).

Gender Chi^2^ –P = 0.02

Ethnicity Chi^2^ –P<0.0001

HbA1c C vs T1DM (P<0.0001), C vs DSPN (P<0.0001), T1DM vs DSPN (NS).

T-CHL C vs T1DM (P = 0.006), C vs DSPN (P = 0.002), T1DM vs DSPN (NS).

ACR C vs T1DM (NS), C vs DSPN (P = 0.004), T1DM vs DSPN (P = 0.03).

eGFR C vs T1DM (NS), C vs DSPN (NS), T1DM vs DSPN (P = 0.006).

Table key

ACR–Albumin Creatinine Ratio, BMI–Body Mass Index, BP–Blood Pressure, C–Controls, estimated Glomerular Filtration Rate, HbA1c –Glycated Haemoglobin A1c, T-CHL–Total Cholesterol, HDL–High Density Lipoprotein Cholesterol.

### Symptoms and deficits (Table 2)

Measures of sign(s) and symptoms of neuropathy are detailed in Table 2. The NDS was significantly greater in patients with DSPN compared to control subjects (P<0.0001) and T1DM without DSPN (P<0.0001), with no significant difference between controls and T1DM without DSPN. The NSP was significantly higher in patients with DSPN (P<0.0001) compared to control subjects and T1DM without DSPN (P<0.0001). The McGill pain score and McGill VAS were significantly greater in diabetic patients with DSPN compared with control subjects (P = 0.001 and P = 0.0007 respectively) and patients with T1DM without DSPN (P = 0.01 and P = 0.02 respectively). There were no differences in NDS, NSP, McGill pain score and VAS between controls and T1DM without DSPN.

**Table 2:** Neuropathy symptoms and deficits in control subjects and diabetic patients without (T1DM) and with (DSPN) neuropathy, with statistically significant differences between groups.

	C (n = 27)	T1DM (n = 30)	DSPN (n = 31)	*T1DM v DSPN*
**NDS (-/10)**	**0.4±0.8**	**1.2±2.0**	**4.6±3.3**	**<0.0001**
**Median (IQR)**	**0 (0–1)**	**0 (0–2)**	**5 (2–7)**	
**NSP (-/38)**	**0.1±0.4**	**1.3±2.0**	**5.0±6.2**	**<0.0001**
**Median (IQR)**	**0 (0–0)**	**0 (0–2)**	**2.5 (0–6)**	
**VAS (-/10cm)**	**0.2±1.0**	**1.0±2.3**	**3.3±3.8**	**0.01**
**Median (IQR)**	**0 (0–0)**	**0 (0–0)**	**0 (0–0)**	
**McGill Pain score**	**0.1±0.4**	**1.9±6.5**	**4.2±6.5**	**0.02**
**Median (IQR)**	**0 (0–0)**	**0 (0–0)**	**2 (0–5)**	

Post Hoc Analyses

NDS: C vs T1DM (NS), C vs DSPN (P<0.0001), T1DM vs DSPN (P<0.0001)

NSP: C vs T1DM (NS), C vs DSPN (P<0.0001), T1DM vs DSPN (P<0.0001)

McGill VAS: C vs T1DM (NS), C vs DSPN (P = 0.001), T1DM vs DSPN (P = 0.01)

McGill Pain score: C vs T1DM (NS), C vs DSPN (P = 0.0007), T1DM vs DSPN (P = 0.02)

Table key

C–Controls, McGill VAS–McGill Visual Analogue Score, NDS–Neuropathy Disability Score, NSP–Neuropathy Symptom Profile.

### Neuropathy evaluation (Table 3)

Non–contact corneal aesthesiometery, small and large fibre neuropathy measures are highlighted in Table 3.

**Table 3:** Small and large fibre tests of nerve structure and function in control subjects and diabetic patients without (T1DM) and with (DSPN) neuropathy, with statistically significant differences between groups.

	C (n = 27)	T1DM (n = 30)	DSPN (n = 31)	*T1DM v DSPN*
**NCCA (mBar)**	0.5±0.3	0.8±0.7	1.3±2.6	NS
**CNFD (no/mm^2^)**	**37.2±5.1**	**30.1±6.7**	**19.8±9.2**	**<0.0001**
**CNBD (no/mm^2^)**	**92.0±36.2**	**60.7±27.9**	**45.4±32.0**	**0.02**
**CNFL (mm/mm^2^)**	**26.6±3.8**	**21.5±4.8**	**15.8±7.0**	**0.001**
**IENFD (no/mm)**	**10.2±3.3**	**8.3±5.5**	**4.7±4.3**	**0.001**
**CST (°C)**	**28.6±2.0**	**27.5±2.0**	**21.4±9.1**	**0.0007**
**WST(°C)**	**36.4±2.0**	**38.1±3.4**	**41.8±4.5**	**0.0004**
**VPT (volts)**	**5.3±4.1**	**5.6±2.5**	**18.4±12.2**	**<0.0001**
**Sural SNCV (m/s)**	**50.6±2.0**	**47.1±4.1**	**39.4±6.1**	**<0.0001**
**Sural Amp (μV)**	**20.2±8.8**	**15.1±6.1**	**5.5±4.2**	**<0.0001**
**Peroneal MNCV (m/s)**	**49.2±3.7**	**45.5±2.2**	**35.4±8.6**	**<0.0001**
**Peroneal Amp (mV)**	**6.1±2.4**	**7.3±9.7**	**2.4±2.1**	**<0.0001**

7.3±9.7

6.1±2.4

Peroneal Amp (mV)

Post hoc analyses

CNFD: C vs T1DM (P<0.0001), C vs DSPN (P<0.0001), T1DM vs DSPN (P<0.0001)

CNBD: C vs T1DM (P = 0.0008), C vs DSPN (P<0.0001), T1DM vs DSPN (P = 0.02)

CNFL: C vs T1DM (P<0.0001), C vs DSPN (<0.0001), T1DM vs DSPN (P = 0.001)

IENFD: C vs T1DM (P = 0.02), C vs DSPN (P<0.0001), T1DM vs DSPN (P = 0.001)

CST: C vs T1DM (NS), C vs DSPN (P<0.0001), T1DM vs DSPN (P = 0.0007)

WST: C vs T1DM (NS), C vs DSPN (P<0.0001), T1DM vs DSPN (P = 0.0004)

VPT: C vs T1DM (NS), C vs DSPN (P<0.0001), T1DM vs DSPN (P<0.0001)

SNCV: C vs T1DM (P = 0.0008), C vs DSPN (P<0.0001), T1DM vs DSPN (P<0.0001)

SNAmp: C vs T1DM (NS), C vs DSPN (P<0.0001), T1DM vs DSPN (P<0.0001)

PMNCV: C vs T1DM (P<0.0001), C vs DSPN (P<0.0001), T1DM vs DSPN (P<0.0001)

PMNAmp: C vs T1DM (NS), C vs DSPN (P<0.0001), T1DM vs DSPN (P<0.0001)

Table key

C–Controls, CNFD–Corneal Nerve Fibre Density, CNBD–Corneal Nerve Branch Density, CNFL–Corneal Nerve Fibre Length, CST–Cold Sensation Threshold, IENFD–Intra Epidermal Nerve Fibre Density, PMNAmp–Peroneal Motor Nerve Amplitude, PMNCV–Peroneal Motor Nerve Conduction Velocity, SSNAmp–Sural Nerve Sensory Nerve Amplitude, SMNCV–Sural Motor Nerve Conduction Velocity VPT–Vibration Perception Threshold, WST–Warm Sensation Threshold.

#### Electrophysiology

Peroneal nerve conduction velocity was significantly lower in DSPN compared to controls (P<0.0001) and T1DM without DSPN (P<0.0001) and between T1DM without DSN and controls (P<0.0001). Peroneal nerve amplitude was significantly lower in DSPN compared with T1DM without DSPN (P<0.0001) and controls (P<0.0001). Sural nerve conduction velocity and amplitude were significantly lower in DSPN (P<0.0001 and P<0.0001 respectively) compared with control subjects and T1DM without DSPN (P<0.0001 and P<0.0001 respectively). Sural nerve conduction velocity was lower in T1DM without DSPN compared to controls (P = 0.0008). However, values for sural and peroneal nerve conduction velocities and amplitudes were within the normal reference range in T1DM suggesting that there was minimal large fibre deficit in this group.

#### Vibration perception and thermal thresholds

VPT was significantly greater in DSPN compared to T1DM without DSPN (P<0.0001) and control subjects (P<0.0001). CST was significantly greater in DSPN compared to T1DM without DSPN (P = 0.0007) and control subjects (P<0.0001). WST was significantly greater in DSPN compared to T1DM without DSPN (P = 0.0004) and controls (P<0.0001). There were no differences in VPT, CST and WST between controls and T1DM without DSPN.

#### IENFD and CCM

**Fig 1:**
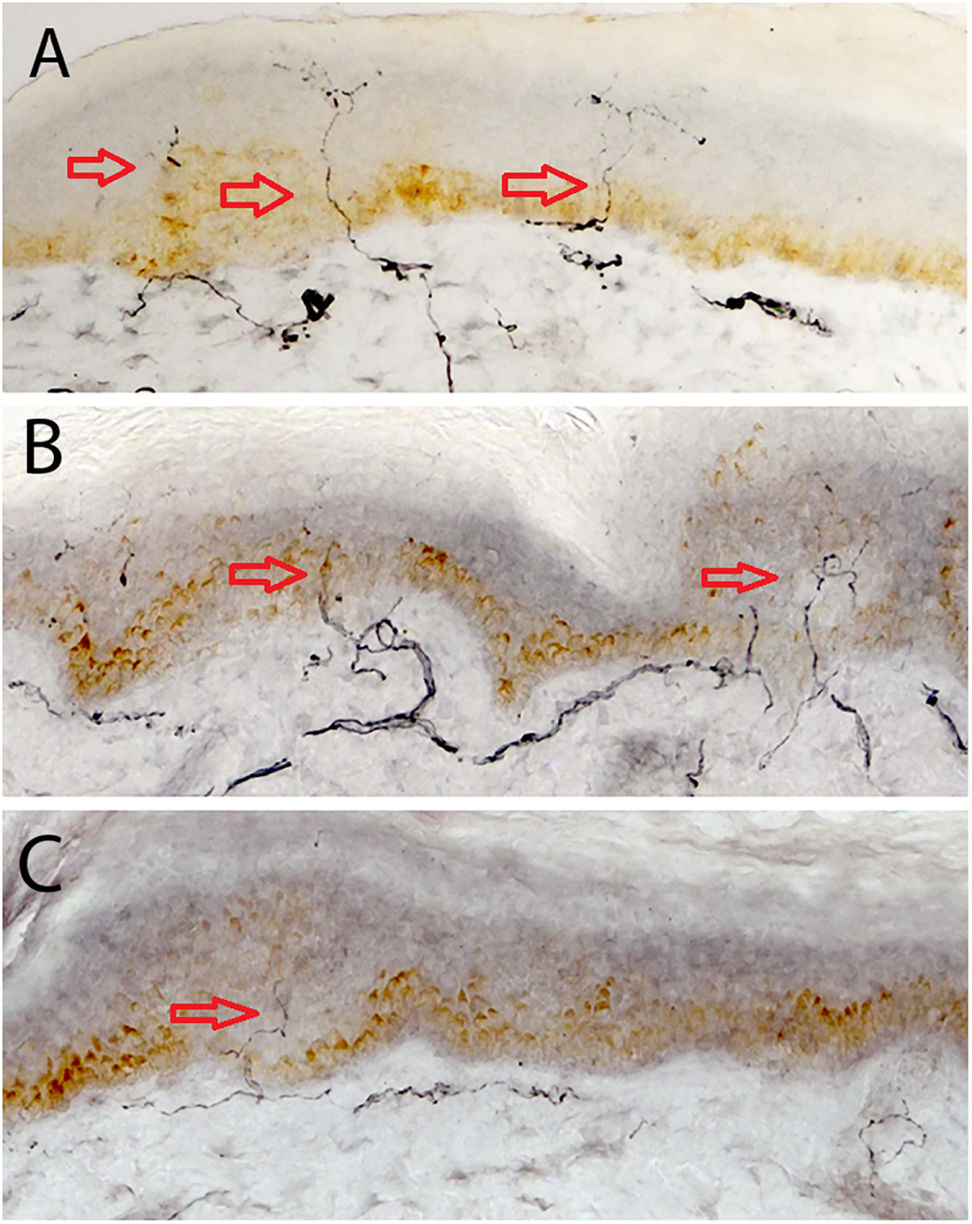
**A, B & C.** Skin biopsy images of IENF in C, T1DM and DSPN.Red arrows point to intra-epidermal nerve fibres.

Fig 1 shows skin biopsy specimens with highlighted IENF in controls, T1DM without DSPN and with DSPN. IENFD was significantly reduced in subjects with DSPN compared to T1DM without DSPN (P = 0.001) and control subjects (P<0.0001) and in T1DM without DSPN compared to controls (P = 0.02). Red arrows point to intra-epidermal nerve fibres.

**Fig 2:**
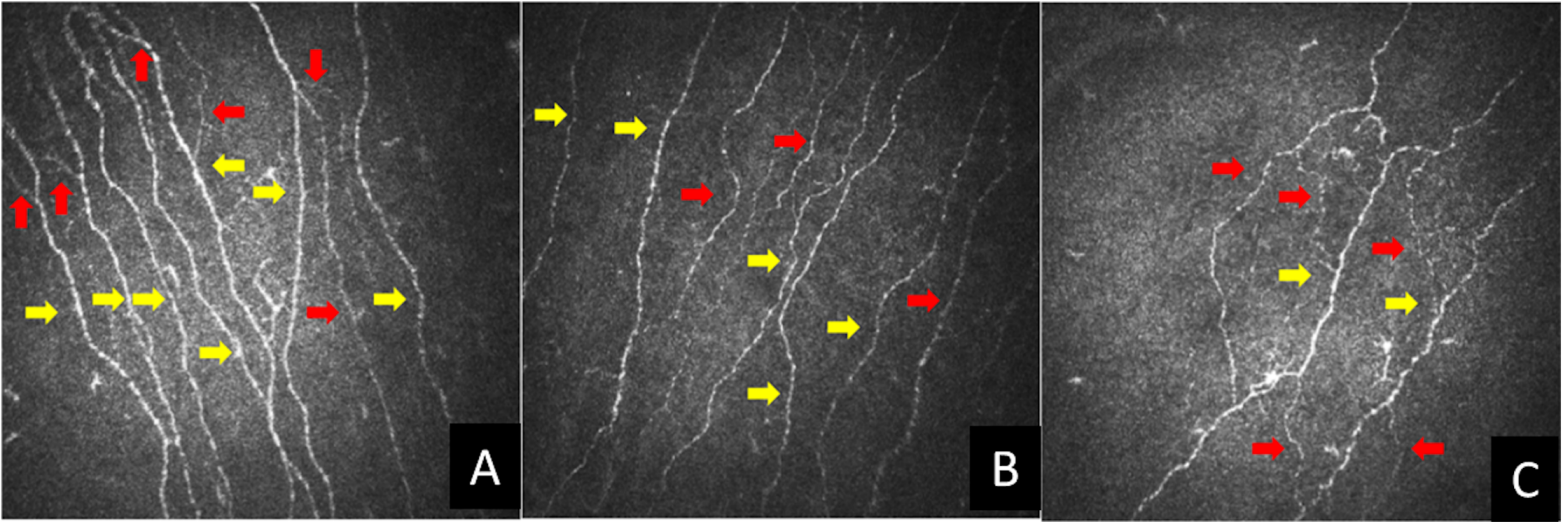
**A, B & C.** CCM images in C, T1DM and DSPN. Red arrows show corneal nerve branches and yellow arrows show corneal nerve fibres.

Fig 2 shows CCM images with highlighted corneal nerves in controls, T1DM without DSPN and with DSPN from the same subjects as in Fig 1. CNFD, CNBD and CNFL were significantly lower in DSPN compared with both T1DM without DSPN (CNFD: P<0.0001, CNBD: P = 0.02 and CNFL: P = 0.001) and controls (CNFD: P<0.0001, CNBD: P<0.0001 and CNFL: P<0.0001). These parameters were also significantly lower in patients with T1DM compared to controls (CNFD: P<0.0001, CNBD: P = 0.0008 and CNFL: P<0.0001) suggesting early small fibre damage. Red arrows show corneal nerve branches and yellow arrows show corneal nerve fibres.

### Correlates of CCM & IENFD (Table 4)

To explore the relationship of CCM parameters and IENFD with other diagnostic modalities, Spearman’s rank correlations of these measures were undertaken and are highlighted in Table 4. The strongest correlation was between CNFD and peroneal motor nerve conduction velocity (Rho = 0.60, P<0.0001). Other neurophysiology measures correlated well with CNFD (peroneal nerve amplitude: Rho = 0.52 P<0.0001, sural nerve velocity: Rho = 0.52 P<0.0001, sural nerve amplitude: Rho = 0.48 P<0.0001). IENFD correlated less well (peroneal motor nerve velocity Rho = 0.56 P<0.0001, peroneal nerve amplitude: Rho = 0.45 P<0.0001, sural nerve velocity: Rho = 0.45 P<0.0001, sural nerve amplitude: Rho = 0.50 P<0.0001). Only a low to moderate correlation (Rho = 0.3–0.49) was found between CCM parameters and IENFD (CNFD: Rho = 0.33 P = 0.001, CNBD: Rho = 0.33 P = 0.003, CNFL: Rho = 0.32 P = 0.002). However, both signs (NDS) and in particular symptoms (NSP and McGill visual analogue score) correlated better with CNFD and IENFD.

**Table 4:** Spearman’s rank correlation of CNFD, CNBD, CNFL and IENFD versus NDS, McGill VAS, NSP, IENFD, thermal thresholds, VPT and nerve conduction studies.

	CNFD	CNBD	CNFL	IENFD
**NDS (-/10)**	• Rho = -0.45 • P<0.0001	• Rho = -0.27 • P = 0.01	• Rho = -0.34 • P = 0.001	• Rho = -0.47 • P<0.0001
**VAS(-/10cm)**	• Rho = -0.43 • P<0.0001	• Rho = -0.34 • P = 0.002	• Rho = -0.43 • P<0.0001	• Rho = -0.45 • P<0.0001
**NSP (-/38)**	• Rho = -0.51 • P<0.0001	• Rho = -0.28 • P = 0.009	• Rho = -0.39 • P = 0.0002	• Rho = -0.51 • P<0.0001
**IENFD (no/mm)**	• Rho = 0.33 • P = 0.001	• Rho = 0.31 • P = 0.003	• Rho = 0.32 • P = 0.002	• N/a
**CST (°C)**	• Rho = 0.37 • P = 0.0005	• Rho = 0.23 • P = 0.04	• Rho = 0.26 • P = 0.02	• Rho = 0.33 • P = 0.002
**WST(°C)**	• Rho = -0.39 • P = 0.0003	• Rho = -0.35 • P = 0.0009	• Rho = -0.33 • P = 0.002	• Rho = -0.52 • P<0.0001
**VPT (volts)**	• Rho = -0.49 • P<0.0001	• Rho = -0.31 • P = 0.004	• Rho = -0.37 • P = 0.0004	• Rho = -0.47 • P<0.0001
**SSNCV (m/s)**	• Rho = 0.52 • P<0.0001	• Rho = 0.40 • P = 0.0002	• Rho = 0.43 • P<0.0001	• Rho = 0.45 • P<0.0001
**SSNAmp (μV)**	• Rho = 0.48 • P<0.0001	• Rho = 0.28 • P = 0.01	• Rho = 0.34 • P = 0.002	• Rho = 0.50 • P<0.0001
**PMNCV (m/s)**	• **Rho = 0.60** • **P<0.0001**	• **Rho = 0.46** • **P<0.0001**	• **Rho = 0.54** • **P<0.0001**	• **Rho = 0.56** • **P<0.0001**
**PMNAmp (mV)**	• Rho = 0.52 • P<0.0001	• ho = 0.40 • P = 0.0002	• Rho = 0.52 • P<0.0001	• Rho = 0.45 • P<0.0001

The strongest correlations are for CCM parameters and IENFD are highlighted in bold.

Table key

CNFD–Corneal Nerve Fibre Density, CNBD–Corneal Nerve Branch Density, CNFL–Corneal Nerve Fibre Length, CST–Cold Sensation Threshold, IENFD–Intra Epidermal Nerve Fibre Density, NSP–Neuropathy Symptom Profile, PMNAmp–Peroneal Motor Nerve Amplitude, PMNCV–Peroneal Motor Nerve Conduction Velocity, SSNAmp–Sural Nerve Sensory Nerve Amplitude, SMNCV–Sural Motor Nerve Conduction Velocity, VAS–McGill Visual Analogue Score, VPT–Vibration Perception Threshold, WST–Warm Sensation Threshold.

### Receiver-Operating Characteristic (ROC) analysis (Table 5, Fig 3)

**Fig 3:**
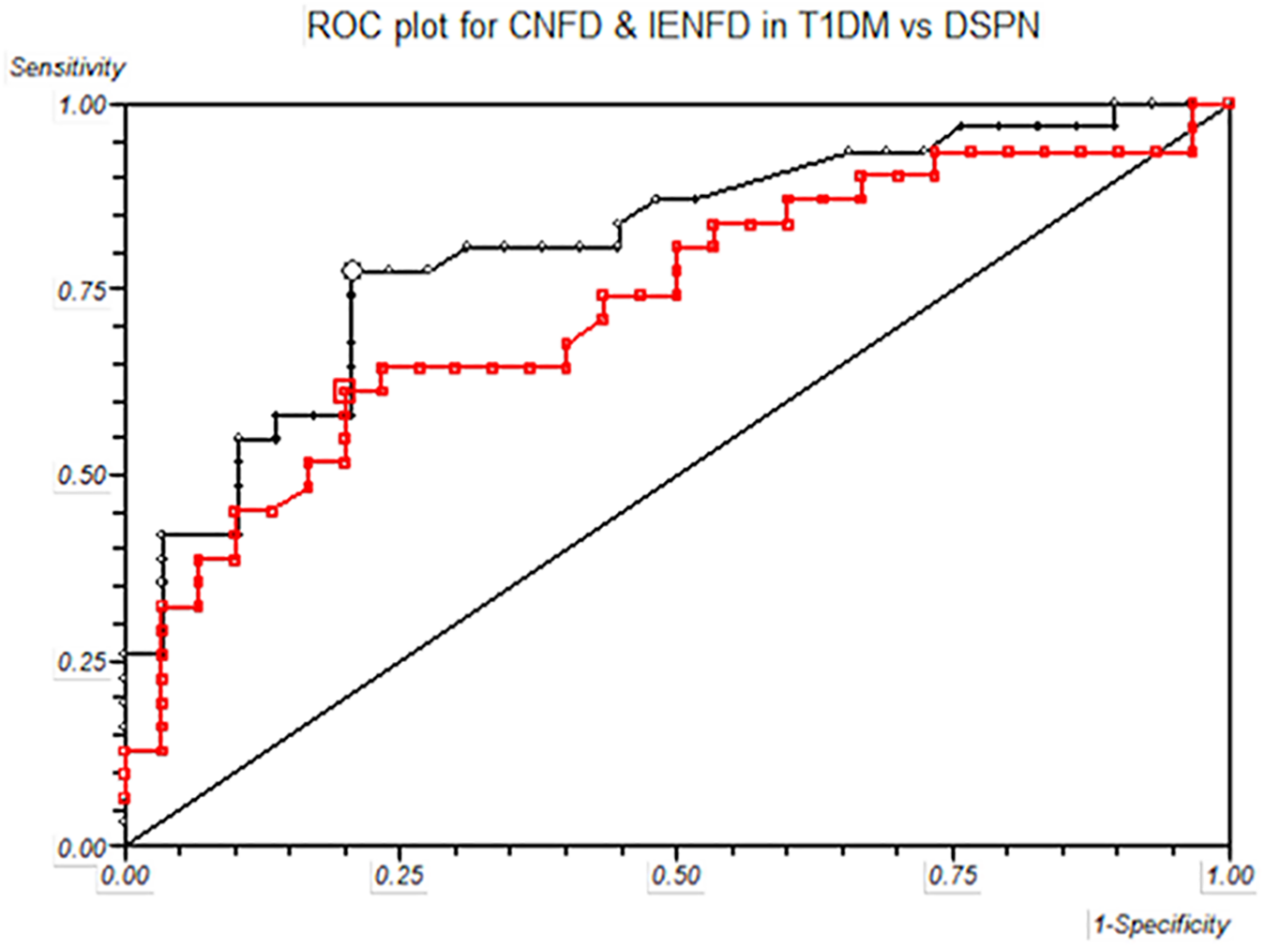
Receiver-operated characteristic (ROC) curves, based on the analysis of CNFD and IENFD in T1DM without DSPN versus with DSPN. Black line represents CNFD and red line represents IENFD.

To assess the diagnostic ability of small and large fibre tests including optimal cut offs, sensitivity and specificity, ROC analysis was undertaken for all measures of neuropathy and are highlighted in Table 5. As the definition of DSPN was based on the nerve conduction studies (Toronto criteria for definite diabetic neuropathy [4]) we have used the ROC analysis for peroneal motor nerve conduction velocity as the referent value for Wilcoxon estimate of area under ROC curve = 0.98 (95% CI: 0.49–1), sensitivity = 0.94 (95% CI: 0.79–0.99) and specificity = 1 (95% CI: 0.88–1). The Wilcoxon estimate of area under the ROC curve was greatest for VPT at 0.85 with an optimal cut off of 13 volts, sensitivity of 0.67, specificity of 1, PPV of 1.0 and NPV 0.75. The small fibre test with the greatest Wilcoxon estimate of area under the ROC curve was CNFD at 0.81 (Fig 3). The PPV and NPV for CNFD were 0.8 and 0.77 respectively for a cut off of 25. IENFD had a lower Wilcoxon estimate of area under the ROC curve at 0.73, which was similar to CNFL (0.74), CST (0.76) and WST (0.74). The PPV and NPV were also lower for IENFD at 0.76 and 0.67 respectively, for a cut off of 4.5 fibres/mm. Black line represents CNFD and red line represents IENFD.

**Table 5:** ROC analysis with area under the curve, optimal cut off and respective sensitivity and specificity with 95% confidence interval in T1DM without DSPN versus DSPN for CNFD, CNBD, CNFL, IENFD, VPT, CST and WST.

	Optimal Cut off	AUC (95% CI)	Sensitivity (95% CI)	Specificity (95% CI)
**CNFD (no/mm^2^)**	**25.0**	• **0.81** • **(0.47–1.0)**	• **0.77** • **(0.59–0.90)**	• **0.79** • **(0.60–0.92)**
**CNBD (no/mm^2^)**	36.5	• 0.67 • (0.45–0.90)	• 0.58 • (0.39–0.75)	• 0.79 • (0.60–0.92)
**CNFL (mm/mm^2^)**	16.8	• 0.74 • (0.46–1.0)	• 0.61 • (0.42–0.78)	• 0.86 • (0.68–0.96)
**IENFD (no/mm)**	**4.5**	• **0.73** • **(0.46–1.0)**	• **0.61** • **(0.42–0.78)**	• **0.80** • **(0.61–0.92)**
**VPT (Volts)**	13	• 0.85 • (0.74–0.95)	• 0.67 • (0.47–0.83)	• 1 • (0.88–1)
**CST (°C)**	24.7	• 0.76 • (0.46–1.0)	• 0.57 • (0.37–0.76)	• 0.89 • (0.72–0.98)
**WST (°C)**	38.0	• 0.74 • (0.61–0.88)	• 0.86 • (0.67–0.96)	• 0.64 • (0.44–0.81)

Table key

CNFD–Corneal Nerve Fibre Density, CNBD–Corneal Nerve Branch Density, CNFL–Corneal Nerve Fibre Length, CST–Cold Sensation Threshold, IENFD–Intra Epidermal Nerve Fibre Density, VPT–Vibration Perception Threshold, WST–Warm Sensation Threshold

### Sub-analyses of age matched groups (S1 Table)

As there was a significant difference in age between those with DSPN versus controls and T1DM we have further sub-analysed age-matched subjects in controls, T1DM and DSPN (Age, C: 46.2±14.0 vs T1DM 43.2±11.1 vs DSPN: 48.5±12.0 years, P = NS). The duration of diabetes was longer, as expected in DSPN (31.1±12.6 years) compared to T1DM (20.2±12.3 years) (P = 0.01). These subgroups were representative of the complete cohort. There were no significant differences between DSPN compared to T1DM for HbA1c, blood pressure or lipids. Clinical signs and symptoms of neuropathy were greater in DSPN compared to T1DM. There were significant differences between CNFD, CNFL, IENFD, VPT, CPT, WST, peroneal nerve conduction velocity and amplitude and sural nerve conduction velocity and amplitude between T1DM and DSPN. CNFD and IENFD had similar and significant correlations to VPT, peroneal nerve conduction velocity and amplitude and sural nerve conduction velocity and amplitude. These sub-analyses are included in the S1 Table. The Wilcoxon estimate of area under the ROC curve was similar for CNFD, CNFL and IENFD (0.68, 0.69 and 0.68 respectively).

## Discussion

There is a need for surrogate end points of diabetic neuropathy, which accurately detect early disease, quantify disease progression and measure therapeutic response [22]. The current ‘gold’ standard for the diagnosis of neuropathy is neurophysiology, a robust measure that also predicts foot ulceration and mortality in diabetes [23]. Other measures of neuropathy such as neurological assessment are poorly reproducible [2], QST is subjective and more accurate measures such as skin and nerve biopsy are invasive and require specialist analysis [4]. Small fibre neuropathy has direct pathophysiological relevance to the main outcomes of pain and foot ulceration [24] and therefore skin biopsy assessment of IENF has been proposed as a valid measure of diabetic neuropathy [8]. Whilst skin biopsy detects early small nerve fibre damage even when electrophysiology and QST are still normal [5], the use of this test in clinical trials is limited by its invasive nature. CCM is a rapid and readily reiterative technique, which quantifies small nerve fibres non-invasively [25–30]. The major findings of this study in relation to the diagnostic ability of different small fibre tests in diabetic neuropathy are: 1) early subclinical small nerve fibre loss can be detected by CCM and IENFD; 2) CNFD has a comparable diagnostic utility to IENFD in the diagnosis of DSPN; 3) CNFD and IENFD have similar correlation to nerve conduction studies.

Early intervention with improved glycaemic control in type 1 DM can lead to a durable reduction in DPN [31]. Furthermore, the need for early evaluation of subclinical small fibre neuropathy has been demonstrated by Smith et al [6] where lifestyle intervention with diet and exercise in a pre-diabetic neuropathy group lead to cutaneous re-innervation and improved pain. Previous studies have employed ROC curve analysis of IENFD at the distal leg and shown a specificity of 95%-97% and sensitivity of 45%- 80% [14, 32] for small fibre neuropathy but these studies were not specifically in patients with DPN. Recently Nebuchennykh et al [13] in a study of patients with polyneuropathy from varying causes showed a sensitivity of 35% and specificity of 95% using a cut off point of 6.7 fibres/mm for IENFD. In another study by Vlckova-Moravcova et al [11], the diagnostic sensitivity for detecting neuropathy was 80% and the specificity was 82% with an optimal IENFD cut off point of ≤8.8 fibres/mm. Although, ROC curve analysis is a standard and appropriate method for establishing diagnostic validity these studies were flawed as they assessed a disease group against a healthy control population and thus sensitivities and specificities will be inappropriately high and the delineation of the optimal cut off points inaccurate. The need to identify DPN in diabetic subjects should mean that optimal cut off points for neuropathy should be based on data from a population of diabetic patients with and without neuropathy rather than a healthy control population versus DPN. Therefore we have performed the present study in a population of diabetic patients with and without neuropathy using the robust Toronto criteria and utilised a best fit ROC curve analysis to derive optimal cut off points, sensitivities, and specificities to assess the diagnostic validity of CCM measures and IENFD. ROC curve analysis in this study showed that IENFD had a sensitivity of 61% and specificity of 80% at an optimal cut off point of 4.5 fibres/mm. These diagnostic validity measures are clearly lower than in the current published literature [11–13], but we believe are truly representative for DSPN as opposed to small fibre neuropathy of other causes. Using exactly the same population and methods we show that CNFD has a similar sensitivity of 77% and an almost identical specificity of 79% at an optimal cut off point of 25.0 /mm.

Previous studies of IENFD have found either absent or weak correlations with nerve conduction studies [11] and sural nerve action potentials [14, 33, 34]. In the present study CNFD, CNFL and IENFD showed a comparable correlation with peroneal motor nerve conduction velocity and amplitude and sural nerve conduction velocity. These data support the study of Shun et al [12] which showed a significant correlation between IENFD with sural nerve action potential and warm sensation threshold. Thermal thresholds continue to be an important reproducible psychophysical test in evaluating small nerve fibres [3, 35] and our data show that the strongest correlation was indeed between IENFD and WST. For DPN, thermal thresholds have previously shown a sensitivity which ranges from 36%-85% [6, 36, 37]. Sensitivities reported in our study are 57% and 86% respectively for CST and WST. Furthermore, we have shown negative correlations of WST with CCM measures and IENFD, confirming a previous study [36]. Interestingly CNBD, CNFD and CNFL correlated with IENFD but the association was a low one (Spearman’s Rho 0.33, 0.31 and 0.32 respectively). Although both CCM and skin biopsy measure small nerve fibres, the sites of assessment are anatomically distinct. There are no significant variations of IENFD calculated in adjacent sections from the same biopsy or in adjacent biopsies from the same site [14]. However, differences in mean values exist for IENFD between differing sites on the lower limb [14, 38] although no direct correlations have been assessed between sites. Although DPN is considered a length dependent neuropathy, recent studies suggest that lesions may occur in a proximal [39] multifocal fascicular pattern [40]. In the current study, the groups are relatively small, and there are major differences between the T1DM and DSPN groups with regard to age and diabetes duration although these anthropometric differences are typically in keeping with risk factors for diabetic neuropathy. Normative ranges for CNFD, CNFL and IENFD differ depending on the decade of life [41, 42]. Therefore, we have undertaken a further subgroup analyses by age-matching the subjects, which shows comparable results for CNFD, CNFL and IENFD and is representative of the overall study. However, the strength of this study is in the detailed quantification of large and small nerve fibres and the accurate phenotyping of subjects with type 1 diabetes.

The current study provides a robust comparison between IENFD and CCM for the assessment of DPN and confirms the results of previous studies [30, 43]. Both CNFD and IENFD correlated well with clinical signs (NDS), and symptoms (McGill VAS and NSP). Furthermore, CNFD and IENFD had similar diagnostic utility for DPN and comparable correlations with electrophysiology. We believe these data provide a robust platform supporting the use of CCM as a diagnostic test for human diabetic neuropathy.

## Supporting information

S1 Table Spearman’s rank correlation of CNFD, CNFL and IENFD versus VPT and nerve conduction studies in age-matched groups. (DOCX)
