# Supplementary material for: Diagnostic utility of corneal confocal microscopy and intra-epidermal nerve fibre density in diabetic neuropathy
Source: PLoS One. 2017 Jul 18;12(7):e0180175. doi: 10.1371/journal.pone.0180175 (PMC5515394; doi:10.1371/journal.pone.0180175)
Supplement: S1 Table — (DOCX) [file pone.0180175.s001.docx]

S1 Table. Spearman’s rank correlation of CNFD, CNFL and IENFD versus VPT and nerve conduction studies in age-matched groups.

|  | CNFD (n=20) | CNFL (n=20) | IENFD (n=20) |
| --- | --- | --- | --- |
| VPT (volts) | - Rho= -0.38 - P=0.003 | - Rho= -0.28 - P=0.03 | - Rho= -0.36 - P=0.005 |
| SSNCV (m/s) | - Rho= 0.44 - P=0.0008 | - Rho= 0.39 - P=0.003 | - Rho= 0.30 - P=0.02 |
| SSNAmp (µV) | - Rho= 0.37 - P=0.002 | - Rho= 0.26 - P=0.04 | - Rho= 0.43 - P=0.0003 |
| PMNCV (m/s) | - **Rho= 0.55** - **P<0.0001** | - **Rho= 0.54** - **P<0.0001** | - **Rho= 0.46** - **P=0.0003** |
| PMNAmp (mV) | - Rho= 0.47 - P=0.0002 | - Rho= 0.39 - P=0.002 | - Rho= 0.33 - P=0.01 |

The strongest correlations are for CNFD, CNFL and IENFD are highlighted in bold.

**Table key**

CNFD – Corneal Nerve Fibre Density, CNFL – Corneal Nerve Fibre Length, IENFD – Intra Epidermal Nerve Fibre Density, PMNAmp – Peroneal Motor Nerve Amplitude, PMNCV – Peroneal Motor Nerve Conduction Velocity, SSNAmp – Sural Nerve Sensory Nerve Amplitude, SMNCV – Sural Motor Nerve Conduction Velocity VPT – Vibration Perception Threshold, WST – Warm Sensation Threshold.
